# Combination Therapy with Zoledronic Acid and Parathyroid Hormone Improves Bone Architecture and Strength following a Clinically-Relevant Dose of Stereotactic Radiation Therapy for the Local Treatment of Canine Osteosarcoma in Athymic Rats

**DOI:** 10.1371/journal.pone.0158005

**Published:** 2016-06-22

**Authors:** Ryan C. Curtis, James T. Custis, Nicole P. Ehrhart, E. J. Ehrhart, Keith W. Condon, Sara E. Gookin, Seth W. Donahue

**Affiliations:** 1 Microbiology, Immunology and Pathology Department, College of Veterinary Medicine and Biomedical Sciences, Colorado State University, Fort Collins, CO, United States of America; 2 Department of Environmental and Radiological Health Sciences, College of Veterinary Medicine and Biomedical Sciences, Colorado State University, Fort Collins, CO, United States of America; 3 Department of Clinical Sciences, College of Veterinary Medicine and Biomedical Sciences, Colorado State University, Fort Collins, CO, United States of America; 4 Department of Anatomy and Cell Biology, Indiana School of Medicine, Indianapolis, IN, United States of America; 5 Department of Mechanical Engineering, College of Engineering, Colorado State University, Fort Collins, CO, United States of America; Georgia Regents University, UNITED STATES

## Abstract

Clinical studies using definitive-intent stereotactic radiation therapy (SRT) for the local treatment of canine osteosarcoma (OSA) have shown canine patients achieving similar median survival times as the current standard of care (amputation and adjuvant chemotherapy). Despite this, there remains an unacceptable high risk of pathologic fracture following radiation treatment. Zoledronic acid (ZA) and parathyroid hormone (PTH) are therapeutic candidates for decreasing this fracture risk post-irradiation. Due to differing mechanisms, we hypothesized that the combined treatment with ZA and PTH would significantly improve bone healing more than ZA or PTH treatment alone. Using an orthotopic model of canine osteosarcoma in athymic rats, we evaluated bone healing following clinically-relevant doses of radiation therapy (12 Gy x 3 fractions, 36 Gy total). Groups included 36 Gy SRT only, 36 Gy SRT plus ZA, 36 Gy SRT plus ZA and PTH, 36 Gy SRT plus PTH, and 36 Gy SRT plus localized PTH treatment. Our study showed significant increases in bone volume and increased polar moments of inertia (in the distal femoral metaphysis) 8 weeks after radiation in the combined (ZA/PTH) treatment group as compared to radiation treatment alone. Histomorphometric analysis revealed evidence of active mineralization at the study endpoint as well as successful tumor-cell kill across all treatment groups. This work provides further evidence for the expanding potential indications for ZA and PTH therapy, including post-irradiated bone disease due to osteosarcoma.

## Introduction

Osteosarcoma (OSA) is the most common primary bone tumor in dogs, accounting for approximately 85% of all bone cancers [1]. Osteosarcoma is also the most frequently diagnosed primary bone tumor in people and shares many similarities with its canine counterpart [2]. It is a locally aggressive tumor with a high rate of metastasis, typically to the lungs [3]. Radiation therapy is a commonly used local treatment for canine osteosarcoma, and it is most often administered with palliative intent. Palliative protocols aim to decrease pain, inflammation, and lameness, while slowing tumor progression and improving quality of life [4]. Due to the goals of therapy, palliative protocols are well tolerated by normal tissues, but never achieve a biologically effective dose (BED) sufficient for long-term tumor control [5–8]. Definitive-intent radiation therapy, relying solely upon conventional fractionation for normal tissue sparing, also fails to achieve a BED sufficient for local tumor control [9]. However, the precision and accuracy of stereotactic radiation therapy (SRT) spares adjacent, healthy tissues via avoidance, while also allowing for the successful targeting and delivery of a sufficiently higher BED within the tumor. Thus, by achieving significant tumor cell death and maximizing survival time, SRT can be considered for definitive-intent therapy.

At Colorado State University’s Flint Animal Cancer Center, dogs undergoing definitive-intent SRT treatment (3 daily fractions of 12 Gy, 36 Gy total dose) for primary-appendicular-osteosarcoma achieved median survival times similar to those of dogs treated with the current standard of care (amputation and adjuvant chemotherapy) (Custis, JT, June 2014, personal communication). Despite this, there was a high risk of pathologic fracture, related to the tumor, following radiation treatment (~33%). Medical interventions, capable of improving bone healing following radiation exposure, could significantly improve outcomes compared to radiation therapy alone. Not only would the companion animal canine population directly benefit from new therapeutics, but they would potentially serve as a translational model for humans with OSA.

Zoledronic acid (a potent anti-resorptive bisphosphonate) and parathyroid hormone (a bone anabolic agent) are potential therapeutics for this purpose. Zoledronic acid (ZA), a third-generation nitrogen-containing bisphosphonate, demonstrates the strongest inhibitory profile against osteoclasts and the strongest binding affinity in comparison to all other bisphosphonates [10]. Recent studies have also implicated the potential for ZA as a radio-sensitizing agent in both tumoral and non-tumoral cell lines [11–12]. Parathyroid hormone (PTH), when administered intermittently, results in bone formation outpacing bone resorption and increasing bone mass [13–16]. Parathyroid hormone also has been linked to hibernating animals’ ability to maintain bone strength during periods of hibernation [17]. In dystrophin-deficient mice, black bear PTH (bbPTH) had potent anabolic effects on trabecular bone [18] and was nearly twice as potent as human PTH at increasing trabecular bone volume [19]. The anabolic actions of PTH make it an attractive candidate for reducing fracture risk in post-irradiation cancer-induced bone disease.

The aim of this study was to determine the ability of SRT with bbPTH and ZA, used alone or in combination, to improve bone healing in OSA-affected bone in comparison to SRT alone. Based on the preclinical and clinical studies illustrating PTH’s potent anabolic effect on bone, as well as ZA’s ability to inhibit osteoclastic resorption, it was our hypothesis that their combined treatment would result in greater improvements in bone healing, than treatment with either agent alone, following stereotactic radiation therapy for the local treatment of osteosarcoma.

## Materials and Methods

### Animals

Forty immunocompromised, athymic, female nude rats (RH-*Foxn1*^*rnu*^) that were 7 to 8 weeks of age were obtained from the National Institutes of Health and housed at a laboratory animal resources facility. Animals were housed 2 per cage with climate-controlled conditions and allowed free access to standard laboratory diet and water. Rats were acclimated for one week prior to initiation of experiments with tumor inoculation. All animal procedures and experiments were carried out under an approved protocol (# 12-3596A) by the Colorado State University Institutional Animal Care and Use Committee. All inoculation procedures were carried out under isoflurane anesthesia, and all efforts were made to minimize any potential pain and distress through the approved, appropriate use of anesthesia/analgesia.

### Osteosarcoma Cells

Abram’s luciferase-expressing canine osteosarcoma cell line was generously provided by Colorado State University’s Flint Animal Cancer Center. Canine OSA cell validation was performed by multiplex PCR using mitochondrial DNA to ensure the cell line was from canine origin and free of contamination [20]. The cells were grown at 37°C with 5% CO_2_ in minimum essential media supplemented with 10% fetal calf serum, 7.5% sodium bicarbonate, minimum essential media essential amino acids, 10mM non-essential amino acids, L-glutamine and an antibiotic-antimycotic (penicillin-streptomycin) (Mediatech Inc. Manassas, VA., Atlas Biologicals, Fort Collins, CO.). Cells were split at approximately 90% confluency every 1–3 days. Luciferase activity was confirmed by exposing luciferase expressing OSA cells for 5 minutes to luciferin. Expression was captured using a Xenogen IVIS 100 (Caliper Life Sciences, Hopkinton, MA) at a 30 second exposure with medium binning.

### Drug Therapies

Recombinant bbPTH 1–84 was produced by Proteos (Kalamzoo, MI.) and stored lyophilized from PBS at -80°C. The bbPTH was reconstituted in acidic saline solution (0.15M NaCl and 0.001M HCl) to a concentration of 0.1 μg/μl before subcutaneous administration. For local administration, bbPTH was reconstituted in VetriGel (Royer Biomedical, Frederick, MD) (hydrogel polymer) at a concentration of 2.3 μg/μl. Zoledronic acid (Novartis, Basel, Switzerland) was stored at room temperature and reconstituted in sterile normal saline prior to use.

### Osteosarcoma Cell Inoculation

Rats were anesthetized by gaseous chamber induction with isoflurane (4–5%) and oxygen. Rats were transferred to a heated surgical table and anesthesia was maintained via isoflurane (1–3%) mixed with 100% oxygen administered via facemask and a non-rebreathing anesthesia circuit. As previously described, a 22-gauge needle was inserted into the femur at the level of the trochanteric fossa, advanced distally within the medullary canal with a rotating motion to the distal metaphysis, and then withdrawn [21]. A 1ml syringe was then drawn up with 200 μl of MEM at a concentration of 1 x 10^6^ cells per 50 μl. A new 22-gauge needle was placed on the syringe and held upright for three minutes (needle down) to allow for cell settling. The needle was then advanced to the distal metaphysis and 40μl was slowly injected.

### Experimental Design

Eight tumor-inoculated (day 0) rats were randomly assigned to each of five treatment groups: 36 Gy SRT only, 36 Gy SRT plus ZA, 36 Gy SRT plus ZA and bbPTH (1–84), 36 Gy SRT plus bbPTH (1–84) and 36 Gy SRT plus localized bbPTH treatment. There were no non-treatment controls (tumor-bearing, non-irradiated) as previous work found that tumors consistently led to fracture from 3–6 weeks post-inoculation [21]. Radiographs consistent with distal femoral osteosarcoma were confirmed on day 10 following inoculation and radiation therapy was administered on days 14, 15, and 16. Each fraction consisted of 12 Gy of radiation for a total treatment dose of 36 Gy. This radiation protocol was used for all 40 rats in the study. The rats in the ZA group and the combined ZA/bbPTH group were injected subcutaneously once weekly with ZA (12.5 μg/kg) beginning on day 13 (total ZA dose of 100 μg/kg). The rats in the bbPTH and ZA/bbPTH combined groups were injected with bbPTH (100 μg/kg) subcutaneously daily (5 days per week) beginning on day 17. PTH was administered after radiation treatment to minimize its effects on osteosarcoma cells. The localized bbPTH treatment group received 100 μg bbPTH via intramedullary inoculation on day 17, utilizing the same technique described for tumor inoculation. The bbPTH was reconstituted in VetriGel (hydrogel polymer) at a concentration of 2.3 μg/μl just prior to inoculation. To administer the dose of 100 μg, 44 μl of VetriGel was injected slowly over one minute. All rats were imaged with radiography every two weeks and bioluminescence imaging weekly until loss of expression, and at the day 70 endpoint. Animals were euthanized (exsanguination under general anesthesia) 70 days following tumor inoculation or earlier if they developed severe lameness caused by tumor burden or fracture.

### Bioluminescence Imaging

**Fig 1:**
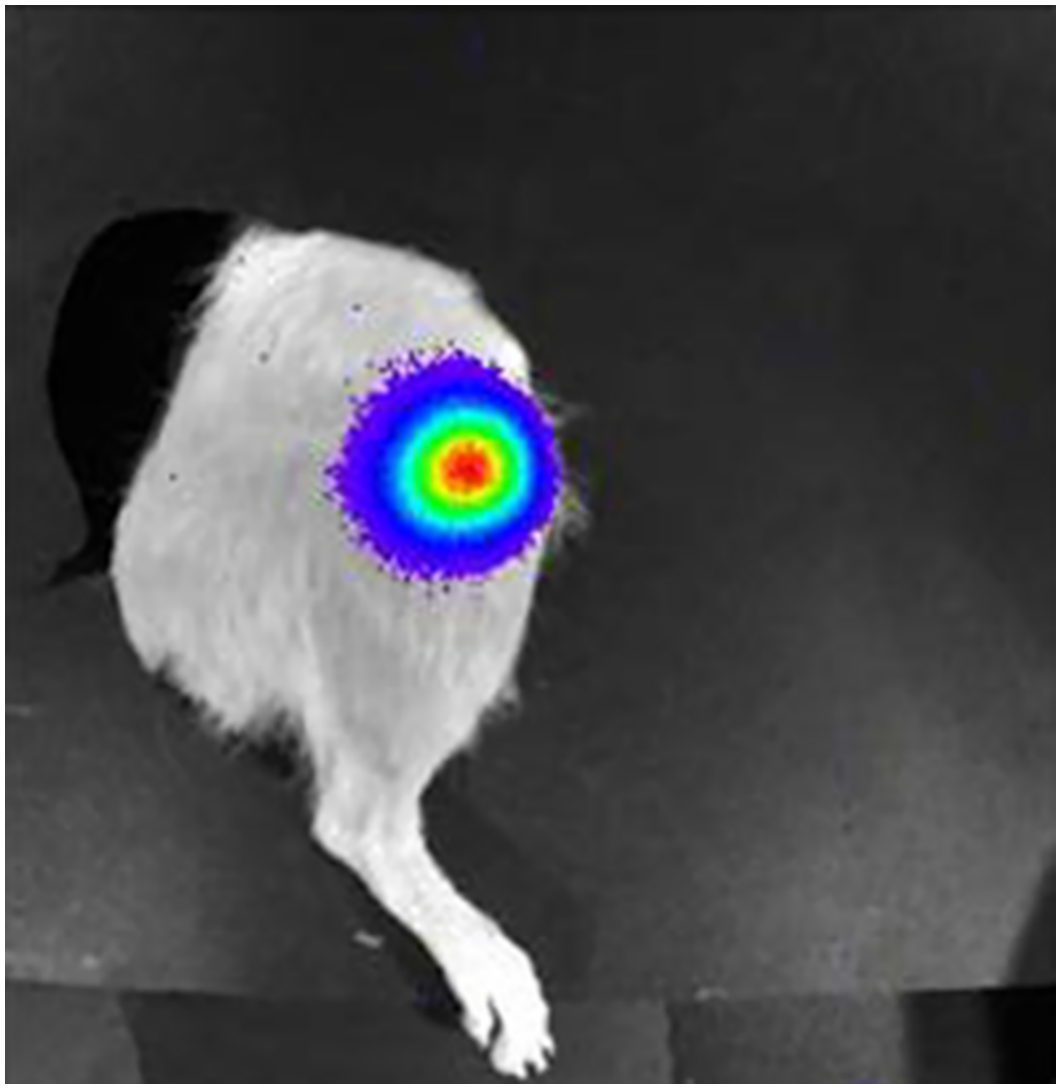
Bioluminescence. Bioluminescence is visualized at the distal femoral metaphysis following tumor cell inoculation.

Rat femurs were imaged on days 0, 4, and 10 and then weekly using a Xenogen IVIS 100 (Caliper Life Sciences, Hopkinton, MA.). All femurs were imaged until complete loss of OSA cell expression and again on the day of sacrifice (day 70). Five minutes prior to imaging, rats were anesthetized by chamber induction (4–5% isoflurane mixed with 100% O_2_), maintained via facemask (1–3% isoflurane mixed with 100% O_2_) and injected intraperitoneally with 150 μl/rat of luciferin (Caliper Life Sciences, Hopkinton, MA.) (30mg/ml). Rats were positioned in right lateral recumbency in the Xenogen unit and images of the left femur were taken at 3 minute time intervals at medium binning (Fig 1).

### Radiography

While rats were anesthetized, digital radiography of the femurs was performed (every two weeks) to monitor the onset of tumor-associated osteolysis, tumor progression, and development of pathologic fracture. Lateral radiographic views were obtained using 50 kV and 2.5 mA at 0.14 seconds, and stored electronically for comparison and analysis.

**Fig 2:**
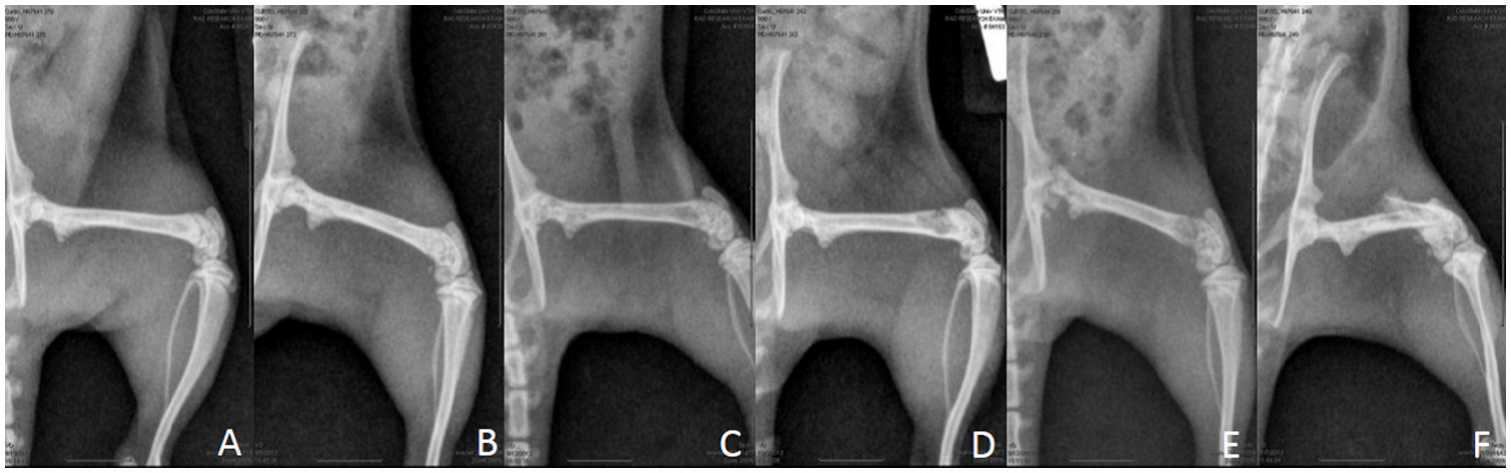
Radiograph severity scoring on a scale from 0–5. A. 0 –No evidence of tumor-associated osteolysis. B. 1 –Mild trabecular lysis apparent (≤ 50% of the diameter of the femur affected. C. 2 –Moderate trabecular lysis apparent (> 50% of the femur’s diameter affected). D. 3 –Severe osteolysis evident with 1 cortex involved. E. 4 –Severe osteolysis evident with both cortices affected. F. 5 –Fracture.

A scoring system was used to monitor the degree of lysis and progression of tumors prior to and throughout the treatment schedule (Fig 2). The evaluator was blinded to treatment group.

### Stereotactic Radiation Therapy

Stereotactic radiotherapy was initiated two weeks after osteosarcoma cell injection and after confirmation of tumor cell engraftment via radiography and bioluminescence imaging. Cone-beam CT (CBCT) images of the affected femur, captured via onboard system and reconstructed with a slice thickness of 1 mm, were imported into the computerized treatment planning system. The gross tumor volume (GTV) was defined to include the entire left femur to ensure dose delivery to all possible tumor cells. For all rats, the clinical target volume (CTV) was identical to the GTV. The planned target volume (PTV) was the result of a symmetric 2-mm expansion beyond the GTV or CTV. An SRT plan consisting of 7 isocentrically placed fields was created. A multi-leaf collimator (5-mm leaf width at isocenter) was used, in a static fashion, to shape each beam to the target volume, minimizing the volume of unaffected tissues within the high dose gradient. Each plan was normalized to achieve a minimum of 99% of the desired dose (36 Gy) within the GTV and a minimum of 95% of the desired dose within the PTV as determined through evaluation of a dose-volume histogram.

Prior to delivery of each SRT fraction, CBCT of the affected femur was obtained with the onboard imaging system. This CBCT was matched to the original, with particular attention made to align the PTV with the affected femur. Any changes in the couch position, based on the matching process, were made to ensure precision and accuracy of SRT delivery.

Rats were anesthetized and underwent a 12 Gy fraction of SRT daily for 3 days for a total treatment dose of 36 Gy. A symmetric 2mm PTV expansion assured inclusion of the GTV through the digital matching of that day’s CBCT with the original used for planning. Quality-control testing of the linear accelerator was performed daily prior to SRT delivery.

### Micro-CT

**Fig 3:**
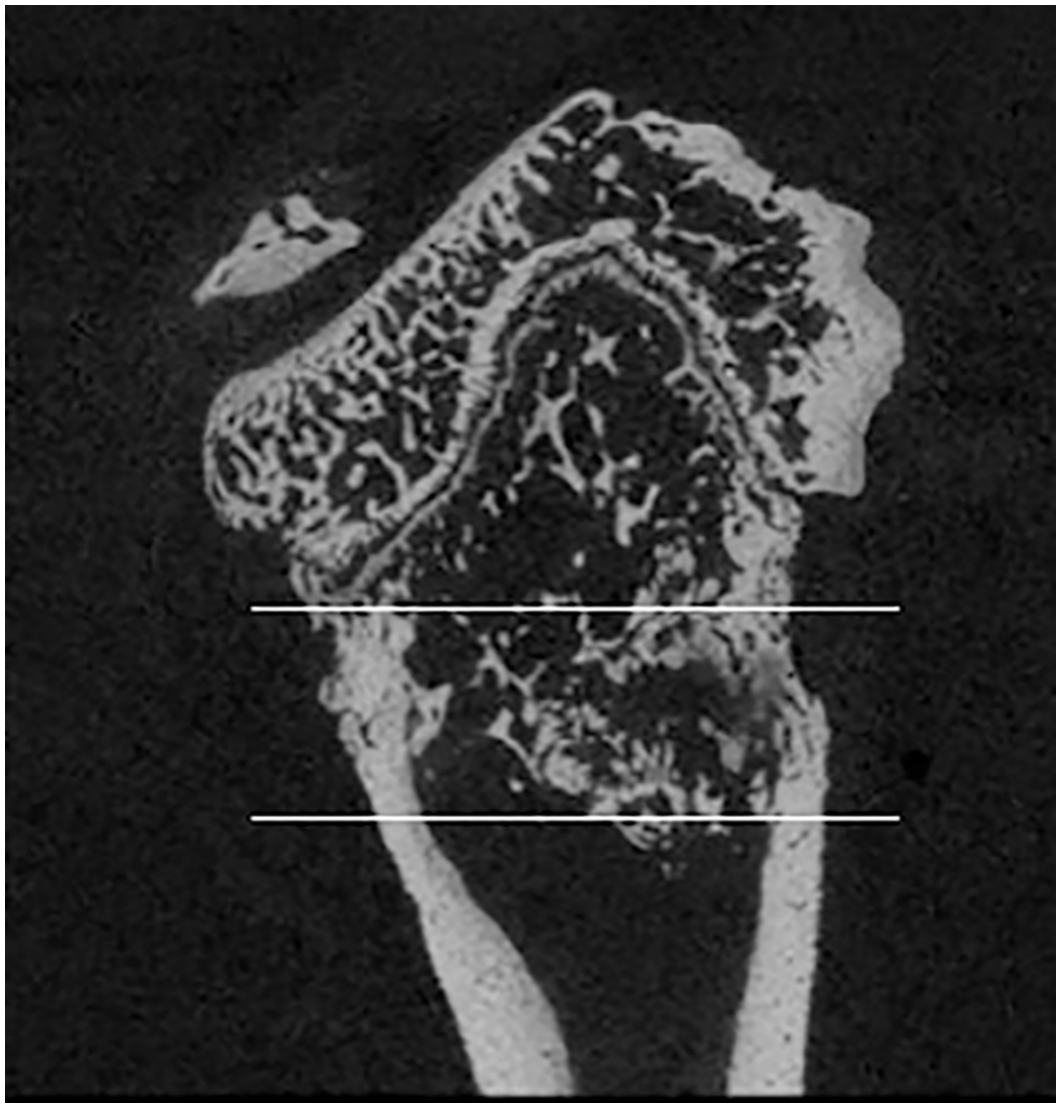
Region of interest for micro-CT analysis.

At the study endpoint on day 70, left femurs (irradiated, tumor-bearing) were collected and soft tissues removed from the osteosarcoma affected limbs. The femurs were isolated and placed in 10% neutral buffered formalin for 48 hours. Then femurs were placed in 70% ethanol until micro-CT scanning. Femurs of all rats were scanned with a Scanco uCT-80 (Scanco Medical AG, Brüttisellen, Switzerland) at 10-micron resolution. The region of interest (ROI) for analyses included the distal femoral metaphysis spanning 3.5 millimeters beginning at the most proximal edge of the distal femoral growth plate (Fig 3). For trabecular bone we quantified bone volume fraction, bone mineral density, trabecular number, trabecular thickness, and trabecular separation. For all bone (including cortical and trabecular) in the ROI, we quantified bone volume and bone mineral density. Polar moments of inertia were also quantified over the entire ROI as a surrogate measure of bone strength. Contralateral (non-irradiated, non-tumor-bearing) femurs were also collected and scanned utilizing the same procedures to assess the systemic effects of drug treatment on normal bones.

### Histology

After micro-CT scanning, non-decalcified femurs were embedded in methylmethacrylate and longitudinally sectioned in the midsaggital-craniocaudal plane. Serial sections were stained with hematoxylin and eosin (H&E), tartrate-resistant acid phosphatase (TRAP), and Von Kossa McNeal’s. Unstained sections were also used to visualize fluorochrome staining (calcein) to quantify double-labeled surfaces and inter-label width (used to calculate mineral apposition rate). Due to the irregular nature of mineralizing bone in the tumor environment, standard dynamic histomorphometric indices were unable to be quantified in much of the ROI. Therefore, fluorochrome labeling was also quantified as a total number of fluorescent pixels in digitized images at 20x magnification in the entire ROI. The calcein was administered at 12 and 3 days prior to euthanasia at a dose of 10 mg/kg given subcutaneously.

Static histomorphometric parameters including TRAP surface, osteoclast numbers and surfaces, osteoid surface, and osteoid thickness were quantified with Bioquant Osteo Software (Bioquant Image Analysis, Nashville, TN). H&E slides were interpreted by an ACVP boarded pathologist (EJE) for the presence and location of osteosarcoma cells as well as interpreting percent tumor necrosis from radiation treatment.

### Statistical Analysis

The micro-CT parameters, radiographic severity scores, and histological parameters were compared between treatment groups with ANOVA and Tukeys post-hoc test (GraphPad Software, Inc. La Jolla, CA). Comparisons with a p-value less than 0.05 were considered significant. Transformations or non-parametric alternatives were used when necessary. Data for polar moment of inertia was transformed using a log transformation due to significant differences in variance. Data for bone resorption markers was analyzed utilizing a Kruskall-Wallis test due to unequal variance.

## Results

### Tumor Development

Thirty-seven of forty rats (93%) inoculated showed positive bioluminescence on days 0, 4, or 10. Evidence of tumor-associated osteolysis from radiographs and CT scans confirmed successful tumor inoculation in all forty rats (100%). All rats that received a radiograph severity score of 1 (mild trabecular osteolysis) were confirmed via on board cone-beam CT scan for evidence of tumor-associated lysis prior to the initiation of radiation treatment.

### Response to Stereotactic Radiation Therapy

#### Bioluminescence Imaging

Bioluminescence was used to monitor tumor regression and recurrence following stereotactic radiation treatment. One rat was euthanized on day 14 due to a displaced fracture. Of the thirty-nine remaining rats, thirty-four lost expression by day 25. Of these remaining five subjects, four lost expression by day 32 and the last subject lost expression by day 39. The loss of bioluminescence is presumed to be due to the success of stereotactic radiation in causing tumor cell death. The precise timeline for cell killing is unknown as radiation resulting in cell death often occurs upon future mitotic events.

Nearly all rats (37/39) showed no return of bioluminescence on the day of sacrifice. Of the two showing a return of bioluminescence, one was at the distal metaphysis (PTH treatment group) and the other near the neck of the femur (local PTH treatment group). Only the tumor at the distal metaphysis was confirmed as recurrence via histology.

#### Radiography

**Fig 4:**
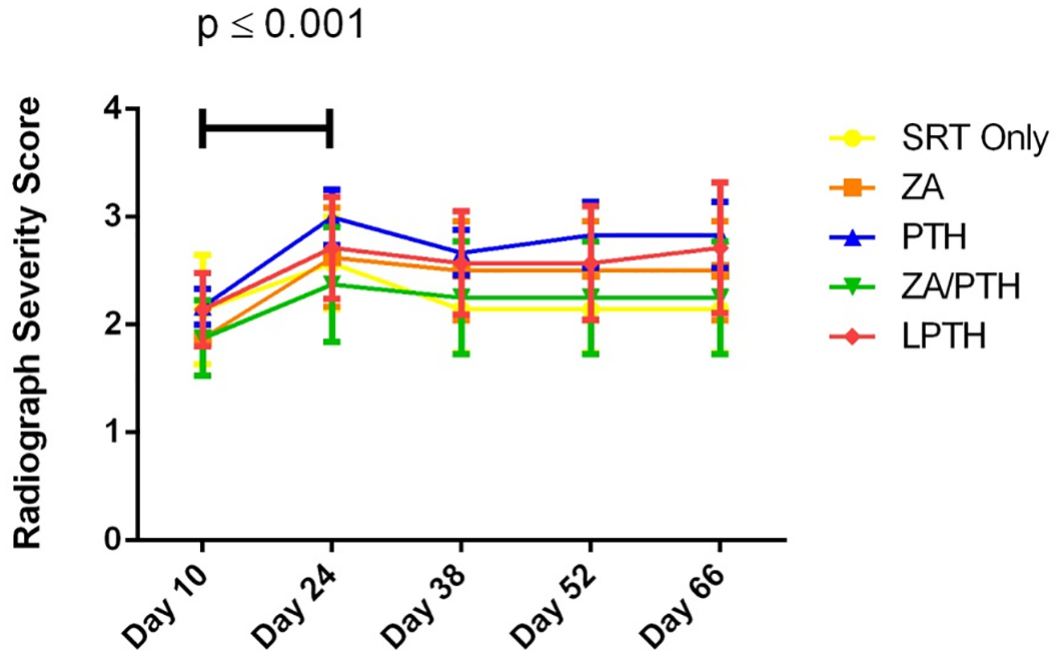
Radiograph severity score over time for each treatment group. There were no significant differences between treatment groups on any given day of imaging. There was a significant difference over all groups from Day 10 to Day 24 (p ≤ 0.001). Score shown as mean +/- SE.

There were no significant differences found between treatment groups on any day that radiographs were taken throughout the experiment. There was a significant (p ≤0.001) increase from the day 10 radiograph score to day 24 in all groups (Fig 4). Following day 24, the radiographic score remained stable for the duration of the experiment, indicating SRT prevented further osteolysis.

#### Fracture

A total of four subjects were euthanized due to fracture prior to the study endpoint of 70 days. These subjects were not included in the statistical analysis of other variables. Upon close evaluation, there appeared to have been severe, early osteolysis (radiograph score of 3 or above) prior to the initiation of treatment and this likely played the most significant role in leading to future fracture on Days 14 (no treatment started), 38 (SRT only), 52 (PTH) and 67 (PTH). Tumor recurrence was not visualized by bioluminescence or histology in any of the subjects that fractured.

#### Micro-CT

**Fig 5:**
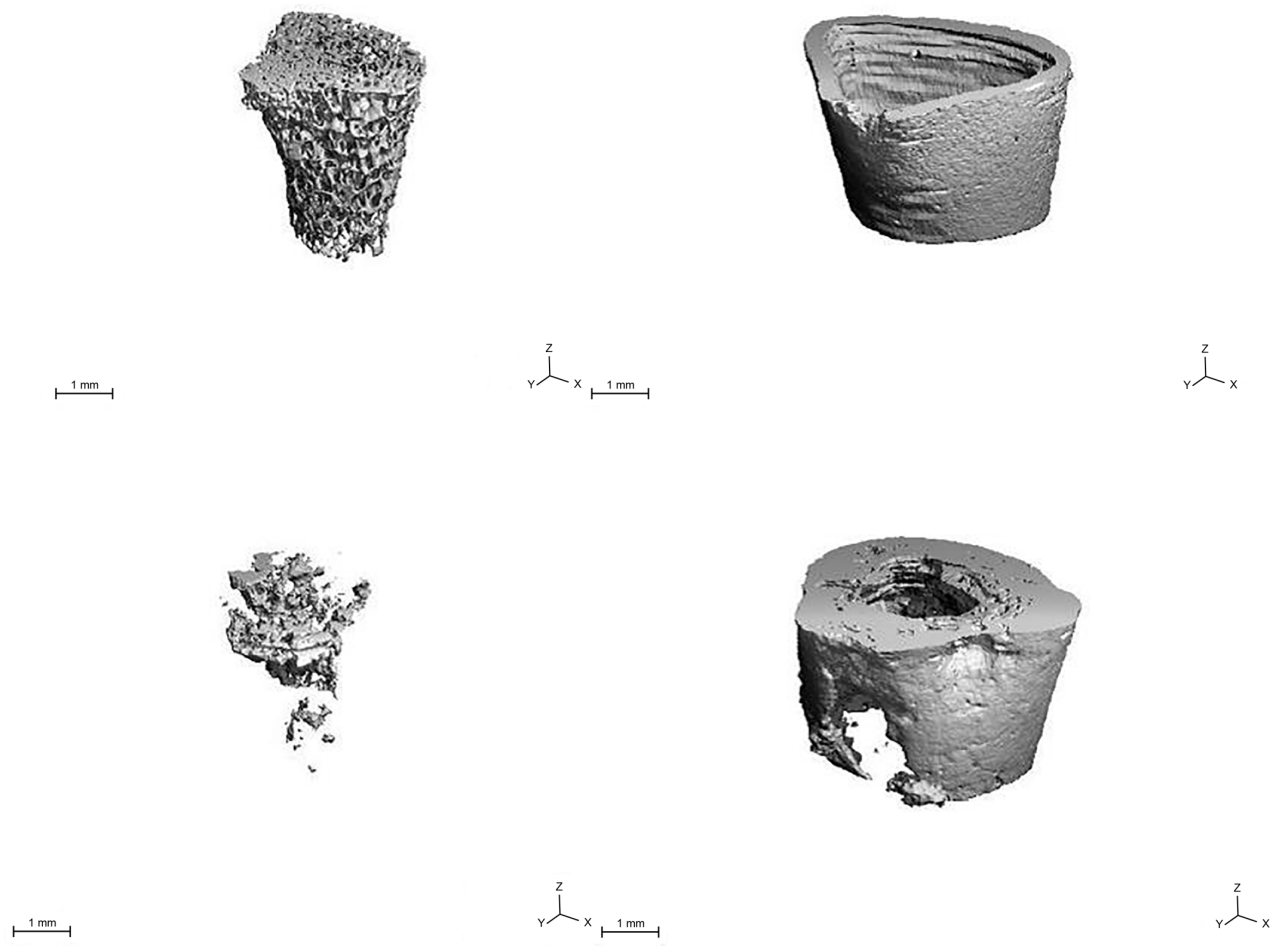
Micro-CT. 3-D reconstructions of normal (top) and tumor-burdened (bottom) trabecular and cortical architecture in the distal metaphysis.

Three-dimensional reconstructions of micro-CT images (distal femoral metaphysis of irradiated, tumor-bearing femurs) revealed significant changes in trabecular architecture including fragmentation of struts and loss of volume. Depending on tumor location, the cortices displayed areas of thinning and loss, however sclerotic areas were also evident (Fig 5).

**Fig 6:**
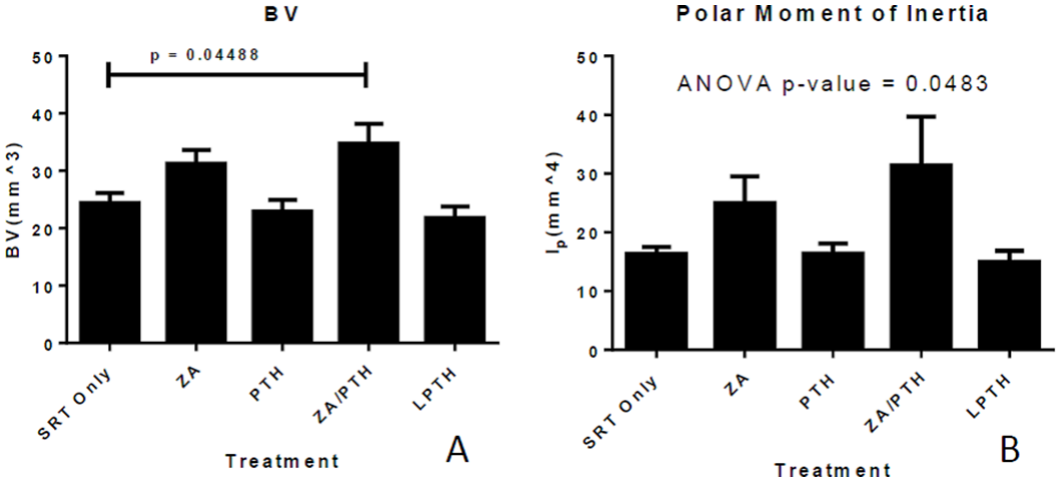
Effects of radiation and adjunct therapy on treated bone. Represented are bone volume (a), and polar moment of inertia (b) in the distal metaphysis of tumor-burdened femora. Data represent mean (+/- SE) values.

There was a significant increase in bone volume (combined total for trabecular and cortical bone) in the combined ZA/PTH treatment group as compared to SRT treatment alone (p = 0.045) (Fig 6a). Measurements of bone mineral density revealed no differences between groups (p > 0.83) for all groups compared to SRT only. Analysis of log transformed data for polar moment of inertia (transformed due to unequal variance) revealed a significant difference between groups (p = 0.0483) (Fig 6b). Despite this, Tukey’s method for multiple comparisons did not result in any significant difference in the pairwise comparisons (p > 0.2). There were no significant differences between treatment groups in any of the variables quantified when only the trabecular compartment was analyzed.

#### Micro-CT Analyses of Contralateral Control Bones

Contralateral femurs (non-irradiated, non-tumor-bearing) were used as relative controls in order to assess the effects of systemic drug treatment on normal bones. Analysis of all bone (cortical and trabecular) over the region of interest revealed significant increases in bone volume in the ZA treated groups (p = 0.0149).

Analysis of the trabecular bone over the region of interest revealed significant increases in bone volume, trabecular number, and trabecular thickness in the ZA treated group compared to controls. Systemic treatment with PTH resulted in significant increases in trabecular thickness and trabecular separation while decreasing trabecular number. Trabecular bone volume was not increased with PTH treatment in controls. These results suggest the increased bone volume seen with combination therapy (ZA/PTH) for the irradiated femurs is primarily due to the ZA treatment. However, it cannot be ruled out that there is some synergistic effect of combination therapy (ZA/PTH), as this was the only group to show statistically significant difference in the irradiated tumor-burdened femurs.

### Dynamic Histomorphometry Reveals Active Mineralization 8 Weeks Post-SRT

**Fig 7:**
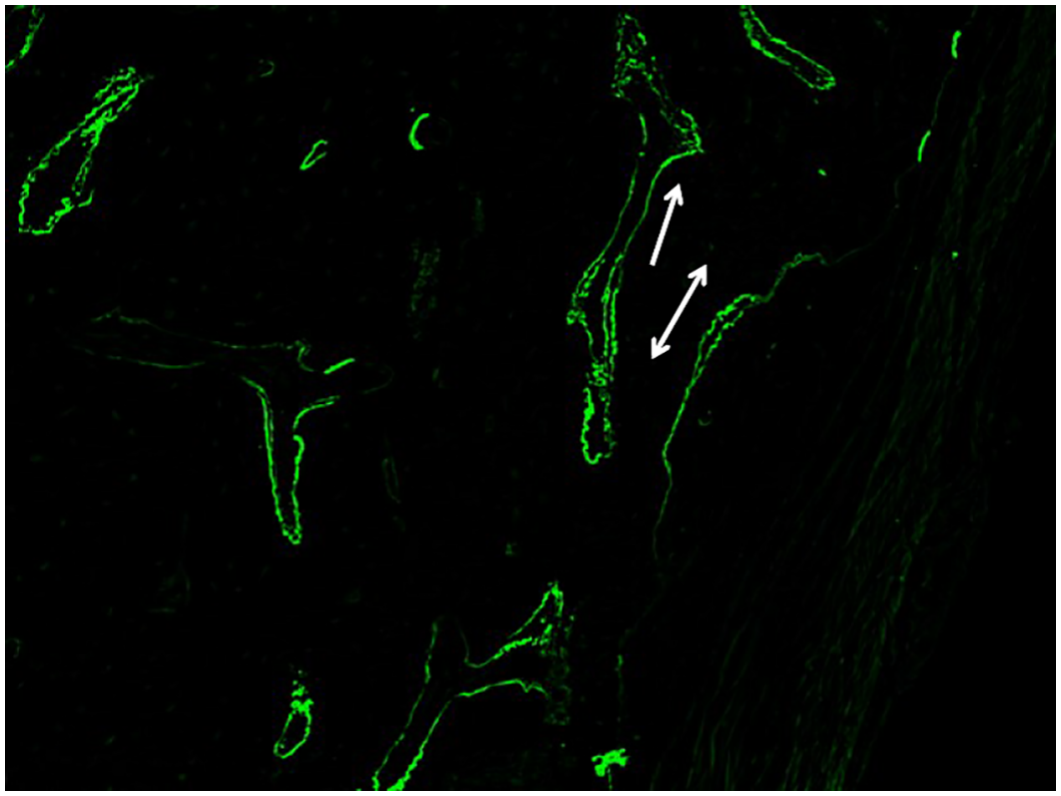
Representative fluorescent micrograph. Image illustrates single (line with one arrowhead) and double-labeled surfaces (line with two arrowheads) in trabecular bone.

Single and double-labeled surfaces were present in the trabecular and cortical bones of all subjects in all treatment groups (Fig 7), indicating active mineralization 8 weeks following radiation doses of 36 Gy to the femur. Conventionally-fractionated radiation has been shown to strongly suppress mineralization, however few studies have shown the effect of clinically-relevant doses of stereotactic radiation on bone mineralization [22].

**Fig 8:**
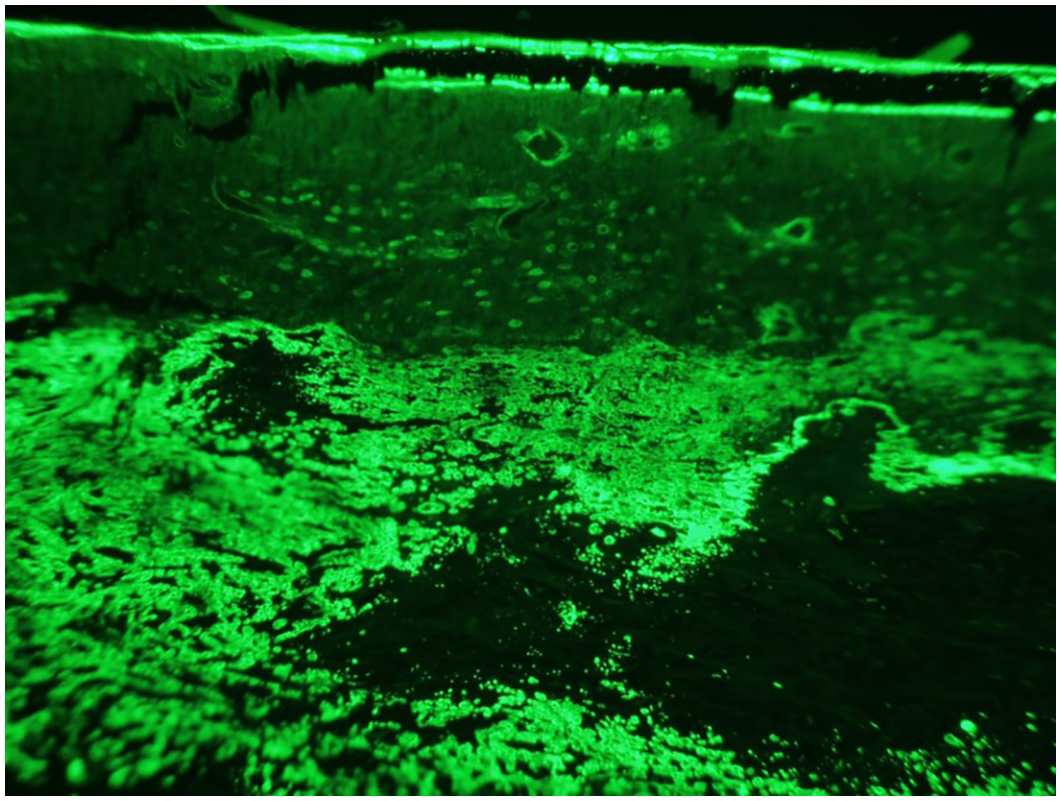
Fluorescent micrograph. Areas of dystrophic mineralization confounded quantification of single-labeled surfaces. As an alternative variable, digital images of the region of interest were used to quantify the total number of positive florescent pixels. There were no significant differences between treatment groups.

Dystrophic mineralization confounded the quantification of single-labeled surface, thus only double-labeled surface and inter-label width were measured (Fig 8). There were no significant differences (p > 0.1504) between treatment groups in regard to double-labeled surface, mineral apposition rate, or total fluorescent pixel number over the region of interest. Although no significant differences were found, the mean mineral apposition rate in experimental groups (SRT Only– 2.23, ZA– 2.66, PTH– 2.88, ZA/PTH– 2.20, LPTH– 2.48 μm/day) was similar to control rats of other studies, suggesting relatively normal rates of mineralization 8 weeks following stereotactic radiation therapy [22, 23]. It should be noted that the percentage of trabecular bone surface comprised of double-labeled surface was quite small likely due to the combination of tumor-associated lysis and radiation effect over the region of interest.

### Static Histomorphometry

#### Tumor Location and Necrosis

**Fig 9:**
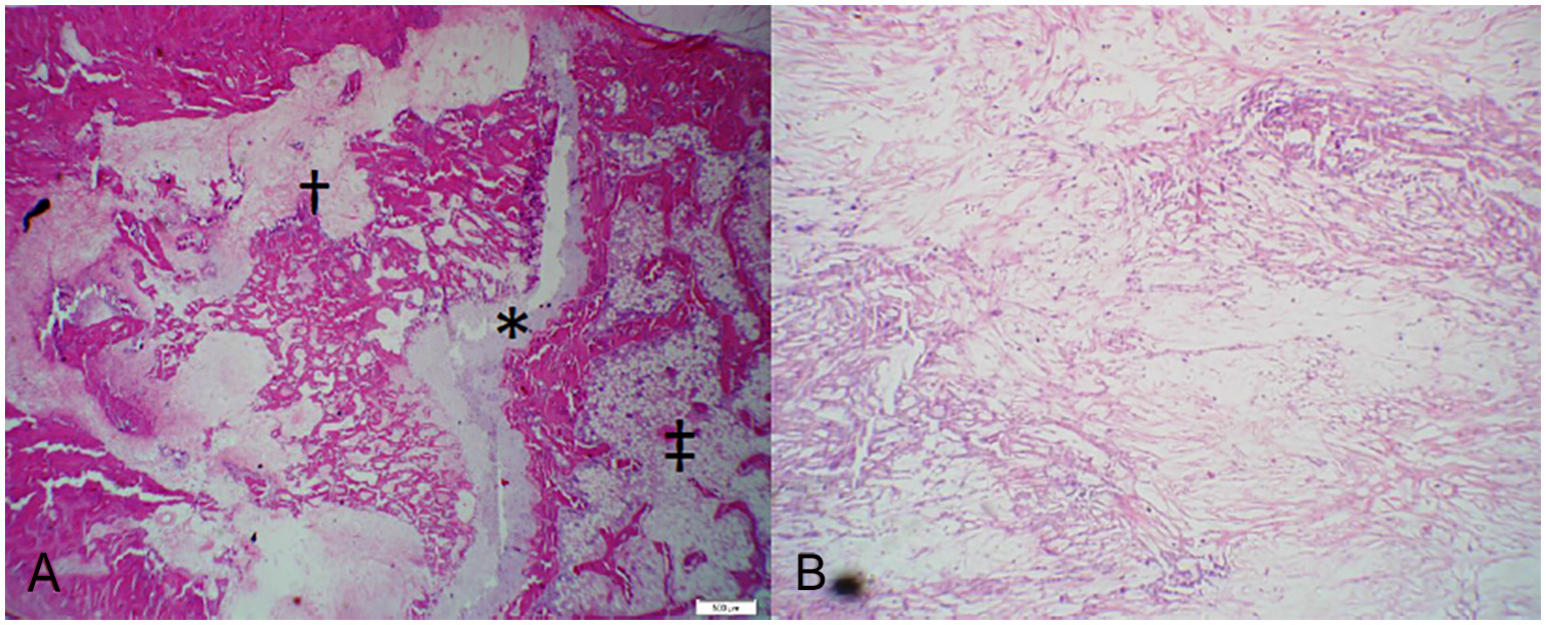
Evidence of previous osteosarcoma. **(H&E)** a) Distal metaphysis exhibiting trabecular destruction, necrosis, and replacement fibrosis throughout regions of previous tumor burden. The growth plate (asterisk), metaphysis (dagger), and epiphysis (double dagger) are shown for reference. (20x) Note the metaphyseal tumor does not cross the growth plate to the epiphysis, where normal trabecular architecture is evident. b) Loosely organized fibrous connective tissue and necrosis in tumor-burdened areas. (100x).

The location of the irradiated osteosarcoma was identified by discrete areas of necrosis with a loosely-defined network of fibrous connective tissue and no viable cells (Fig 9). The distal metaphyseal location of previous tumor burden was confirmed via histologic evaluation in 95% of experimental subjects. The most severe lesions due to osteosarcoma were evident in the distal metaphysis (consistent with imaging) however, evidence of previous osteosarcoma cells in the diaphysis of the femur was confirmed in 90% of experimental subjects. The cells deposited in the diaphysis typically did not result in radiographically evident lysis or fracture but were confirmed present via histology. No experimental subjects had osteosarcoma in the distal epiphysis, highlighting the tumor’s inability to cross the growth plate. One subject did not show evidence of active or previous osteosarcoma over the entire femoral section.

**Fig 10:**
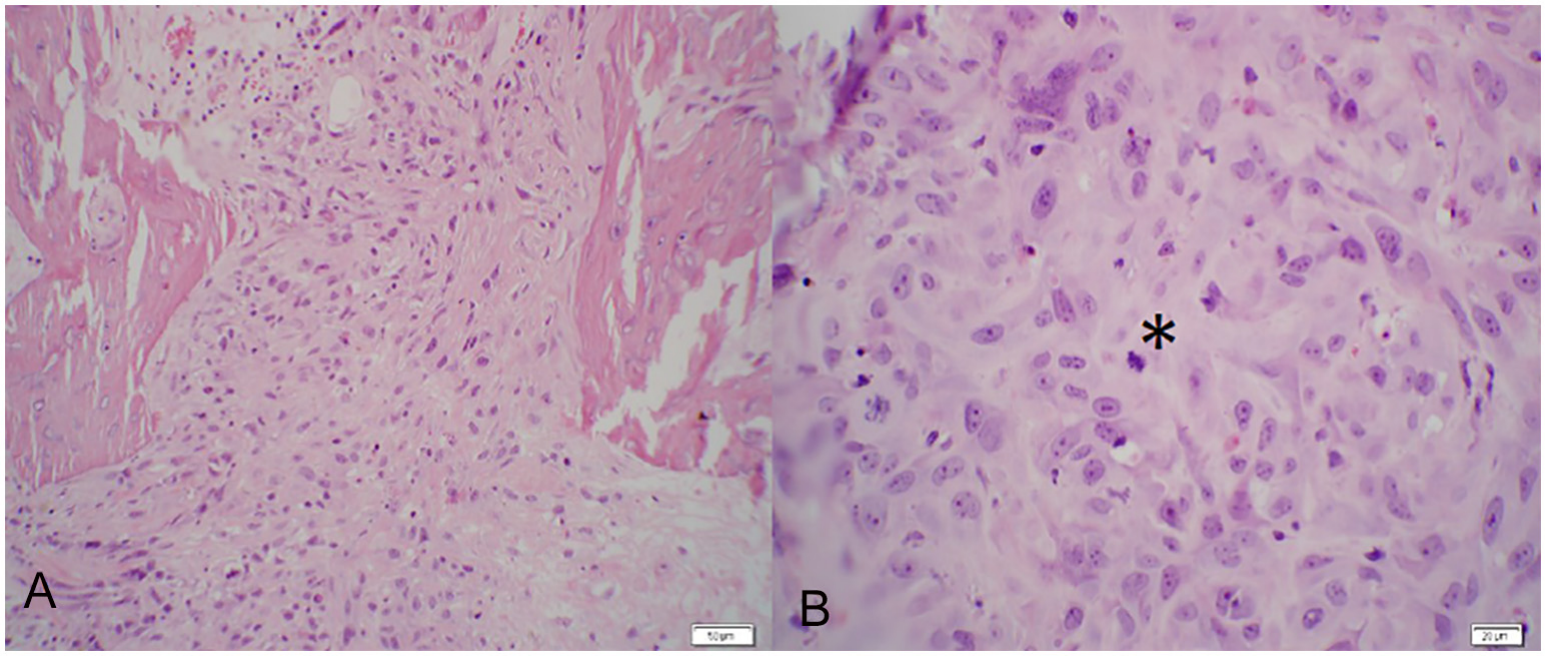
Active Osteosarcoma at the study endpoint. a) Rat. Distal Metaphysis of Femur. (H&E. 200x). Replacing normal trabecular architecture are poorly differentiated neoplastic cells that are polygonal to spindle shaped with lightly eosinophilic cytoplasm. b) Rat. Distal Metaphysis of Femur. (H&E. 400x). Neoplastic cells are irregularly polygonal with indistinct cell borders and moderate amounts of cytoplasm. Nuclei contain multiple, distinct nucleoli and a mitotic figure is present (asterisk).

Tumor necrosis was 100% in thirty-three of thirty-six (92%) experimental subjects. Active osteosarcoma was discovered in four subjects (one from each of the following treatment groups: PTH, LPTH, ZA/PTH, SRT Only), but exhibited an average of 85% tumor necrosis (Fig 10).

#### Tartrate-Resistant Acid Phosphatase

**Fig 11:**
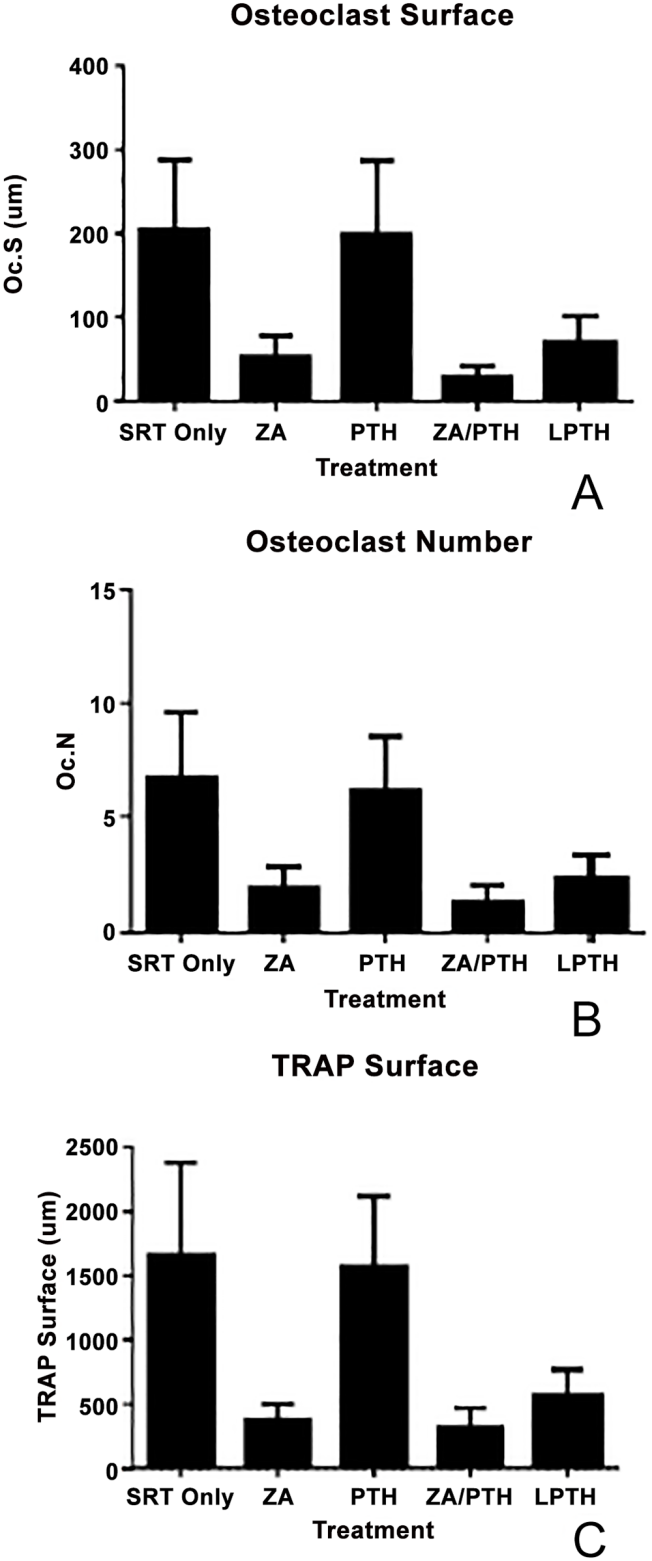
Assessment of bone resorption over the region of interest. (a) There was no difference (p = 0.0627) between groups in osteoclast surface. (b) There was no difference (p = 0.1108) between groups in osteoclast number. (c) TRAP surface showed a statistically significant difference (p = 0.0152) between groups, however, pairwise comparisons (Dunn’s test) was not able to detect which groups.

There were no significant differences between treatment groups for osteoclast number and surface, although the ZA and combined ZA/PTH treatment groups were decreased as expected (ANOVA p-values = 0.1108 and 0.0627 respectively). Analysis of total TRAP surface revealed a difference (p = 0.0152) between groups (Fig 11).

#### Von Kossa McNeal’s

There were no differences in osteoid surface (p = 0.15) or width (p = 0.29) between treatment groups. There were also no differences (p = 0.1) in osteoblast number between groups. The presence of active osteoblasts, similarly to the presence of active osteoclasts, represents the regenerative capabilities of bone 8 weeks post-SRT.

## Discussion

Using an orthotopic model of canine osteosarcoma in athymic rats, we found combination therapy (ZA/PTH) increased bone volume and polar moment of inertia as compared to SRT alone. Polar moment of inertia has been shown to positively correlate with bone strength in nude rats and is a useful surrogate for mechanical properties [24]. Parathyroid hormone and ZA failed to show the same effect when administered alone. Static and dynamic histomorphometry showed there was active bone remodeling and mineralization in all treatment groups 8 weeks after SRT.

Due to their differing mechanisms of action, it has long been hypothesized that the use of combined (ZA/PTH) therapy may result in an additive effect improving bone architecture. Many studies in relation to osteoporosis have failed to show an increased benefit of combination therapy [25–28]. Evidence supporting combination therapy have been shown in relation to disuse osteopenia [29], fracture healing [30], and adjunct treatment following radiation therapy [31]. Although the combination (ZA/PTH) treatment group was the only group to affect bone properties, it appears largely due to the bisphosphonate, zoledronic acid. This is evident by comparing the micro-CT data from control contralateral limbs, which showed increased overall and trabecular bone volume with ZA treatment alone. Despite this, it cannot be ruled out that the combined group had a beneficial therapeutic effect as it was the only group to achieve significant improvements in the experimental tumor-burdened and irradiated femora.

The anabolic effect of intermittently administered PTH has been illustrated in many studies leading to its’ FDA approval for high risk osteoporosis treatment [27, 32, 33]. Bone volume fraction, trabecular number, and trabecular thickness tend to be increased with a decrease in trabecular separation [22–24]. In our study, PTH did not result in these typical changes of bone microarchitecture when administered on its own. However, immunodeficient rodents may have a low osteoanabolic response to intermittent, systemic PTH administration. Nude rats receiving a PTH dose twice as large as what is normally used in rats (80 μg/kg) and administered four times daily (as opposed to daily dosing) resulted in an osteoanabolic response normally seen at lower doses in rats [24]. There is also some evidence supporting the role of T-cells in PTH activity modulation in a mouse model of hyperparathyroidism [34]. Further work is necessary to elucidate the effectiveness of PTH in restoring bone properties following osteolysis due to osteosarcoma and radiation therapy, especially in immunocompetent models.

While PTH has shown its potent anabolic effect in relation to osteoporosis, it has not been approved for use in cancer patients due to the potential increased risk for tumor development, specifically osteosarcoma. Preclinical studies in F344 rats found that a significant percentage of subjects developed osteosarcoma following two years of treatment with PTH [35]. Follow up dose and duration studies discovered that low dose (5 μg/kg) PTH resulted in no neoplastic changes, but high dose (30 μg/kg) PTH administered over 20–24 months (70–80% of lifespan) resulted in significant increases in osteosarcoma incidence [36]. These studies led to the limitation of PTH treatment in humans to 2 years as well as an FDA “black-box” warning. Ongoing surveillance of human osteosarcoma patients has not detected a causal association between PTH therapy and osteosarcoma [37].

Currently in people, bisphosphonates are the first line treatment for osteoporosis and also are used in palliative treatment for metastatic bone disease. From a clinical perspective, ZA is appealing for a number of reasons. Not only does it decrease tumor-related osteolysis, bone pain and fracture risk, but it also exhibits direct and indirect anti-cancer activity [38]. It also has the ability to act as a radiosensitizing agent against tumor cells [11–12]. Zoledronic acid has already been utilized for the palliative treatment of a number of canine osteolytic tumors [39]. ZA administration results in decreased bone resorption markers as well as pain alleviation. Repeated administration of ZA is well tolerated with no evidence of toxicity being identified at a monthly dose of 0.25 mg/kg [39]. A single case report resulted in successful palliation and stable disease for confirmed appendicular osteosarcoma over 16 months. This dog was administered a monthly dose of 4mg that was well-tolerated, reduced pain and improved the quality of life for this large breed dog when other treatment options were not pursued [40].

Stereotactic radiotherapy was delivered successfully and achieved local tumor control in all rats with experimentally-induced osteosarcoma of the distal aspect of the femur. A previous study of radiation therapy for dogs with osteosarcoma showed that a mean percentage tumor necrosis of ≥ 80% correlates with excellent local tumor control and an increase in survival rate [41]. Our study resulted in a mean percentage tumor necrosis of 98%. Active osteosarcoma was found in only four subjects and all of these subjects still maintained tumor necrosis between 80–90%. Previous work with this model showed that if left untreated local progression would lead to fracture around 5 weeks post-inoculation [21]. All of these findings support the positive treatment effect of SRT while highlighting the absence of acute (less than 70 days) clinical side effects during our experimental period.

There are some limitations in this study that need to be addressed. There was variability in tumor severity at the initiation of treatment. Although radiograph severity scoring revealed no significant differences between treatment groups, in vivo micro-CT analysis may provide a more accurate assessment for standardizing tumor severity at the time of treatment. This would allow for an individualized treatment approach relating the starting severity with response to treatment. Secondly, our model proved successful at creating tumor-associated osteolysis at the distal metaphyseal region of the femur, however due to our inoculation technique (proximal to distal) a small number of cells were deposited at more proximal locations of the femur. This typically did not result in visible osteolysis proximally, but their presence was confirmed via histologic examination. Therefore, we included the entire femur for our gross tumor volume in radiation planning regardless if there was evidence or not of osteolysis at other locations of the femur.

Overall, the combined treatment (ZA/PTH) improved bone volume and a geometric surrogate of bone strength following stereotactic radiation therapy in comparison to radiation treatment alone. Our data indicate this result was likely largely due to zoledronic acid, a potent anti-resorptive. However, PTH may have a blunted osteoanabolic response in immunodeficient rats and therefore requires additional research. Zoledronic acid shows promise and potential for decreasing bone pain due to lysis, improving bone microarchitecture and strength, and reducing fracture risk in canine patients undergoing stereotactic radiation therapy for the local treatment of appendicular osteosarcoma. This work also illustrates the success of stereotactic radiation therapy in achieving local tumor control and allowing bone remodeling and mineralization post-radiation. Longitudinal studies of the long term effect of high dose radiation on bone will be important as the clinical benefits of stereotactic radiation therapy expand its application.

## Supporting Information

S1 Datasets Raw Datasets. (XLSX)
